# Retraction Note: Antibiotic growth promoters virginiamycin and bacitracin methylene disalicylate alter the chicken intestinal metabolome

**DOI:** 10.1038/s41598-021-82285-2

**Published:** 2021-01-28

**Authors:** Ujvala Deepthi Gadde, Sungtaek Oh, Hyun S. Lillehoj, Erik. P. Lillehoj

**Affiliations:** 1 Animal Bioscience and Biotechnology Laboratory, Beltsville Agricultural Research Center, Agricultural Research Service, United States Department of Agriculture, Beltsville, MD 20705 USA; 2 Department of Pediatrics, University of Maryland School of Medicine, Baltimore, MD 21201 USA

Retraction of: *Scientific Reports* 10.1038/s41598-018-22004-6, published online 26 February 2018

The Authors have retracted this Article.

After publication, the United States Department of Agriculture (USDA), where the research was performed, identified a gap in the Institutional Animal Care and Use Committee (IACUC) approved protocols for a published research report during its normal oversight and review processes. The broader programmes received all of the necessary approvals; however, a few specific protocols procedures were not included in the program protocols. The research using antibiotic growth promoter treatments described in this article did not receive the appropriate ethical approval.

All Authors agree with the retraction and its wording.

